# Supplementary material for: Tumor-Promoting Activity and Proteomic Profiling of Cisplatin/Oxaliplatin-Derived DAMPs in Cholangiocarcinoma Cells
Source: Int J Mol Sci. 2022 Sep 11;23(18):10540. doi: 10.3390/ijms231810540 (PMC9502173; doi:10.3390/ijms231810540)
Supplement: Supplementary file 1 [file ijms-23-10540-s001.zip › ijms-1880868-supplementary.pdf]

Tumor-promoting activity and proteomic profiling of Cisplatin/Oxaliplatin-derived DAMPs in Cholangiocarcinoma cells

Table S1 Other overexpressed proteins that probably involved with protumor property.

Accession No.	Protein Name	MW (kDa)	Quantitative Intensity			Fold- change		*p*-value	
			Control	CIS	OXA	CIS	OXA	CIS	OXA
Q15109	Advanced glycosylation end product-specific receptor	43	19617	15096600	4193833	770	214	0.0004	0.0018
Q7Z401	C-myc promoter-binding protein	209	1978	7205	11980	4	6	0.5707	0.0001
Q00532	Cyclin-dependent kinase-like 1	42	1443	26424	2343	18	2	0.0001	0.1622
H3BUD3	Cyclin-F	21	1833	188412	18787	103	10	0.0002	0.0000
P42566	Epidermal growth factor receptor substrate 15	99	1418	45899	2485	32	2	0.0010	0.0884
P03372	Estrogen receptor	66	1233	18537	1526	15	1	0.0001	0.2235
P51810	G-protein coupled receptor 143	44	3888	4903	1592	1	0	0.0009	0.0039
Q6LDS6	Granulocyte-colony stimulating factor	1	3011	12503	30801	4	10	0.2205	0.0015
C9JMY1	Insulin-like growth factor-binding protein 2	20	1709	6511200	1946400	3810	1139	0.0005	0.0000
Q96AZ6	Interferon-stimulated gene 20 kDa protein	20	4085	5418	5324	1	1	0.0073	0.1153
K7EJ09	Interleukin enhancer-binding factor 3	14	2111	6307	14138	3	7	0.9879	0.0001
P05113	Interleukin-5	15	2696	18711	4061	7	2	0.0493	0.0591
E5RHY4	MAPK/MAK/MRK overlapping kinase	7	1547	62763	1843	41	1	0.0000	0.4285
O60882	Matrix metalloproteinase-20	54	3586	5898	8207	2	2	0.0390	0.0048
Q6ZN28	Metastasis-associated in colon cancer protein 1	97	2188	8540	12554	4	6	0.0158	0.0001
Q9Y6R4	Mitogen-activated protein kinase kinase kinase 4	182	1523	180786	17945	119	12	0.0029	0.0002
Q8IVH8	Mitogen-activated protein kinase kinase kinase kinase 3	101	9728	23255	8299	2	1	0.0371	0.1771

A6ZIE8	MUC1 isoform M13	11	2004	412170	87591	206	44	0.0000	0.0005
Q8WXI7	Mucin-16	1519	15496	4288900	387987	277	25	0.0000	0.0012
Q7Z5P9	Mucin-19	805	4713	51941	167340	11	36	0.0027	0.0005
Q7Z7M0	Multiple epidermal growth factor-like domains protein 8	303	3011	546850	86577	182	29	0.0004	0.0001
D6RF94	Protein Wnt;Protein Wnt-8a	40	1738	7418	2116	4	1	0.1239	0.0460
Q9NYK1;B2R9N9;D1CS68	Toll-like receptor 7	121	1622	6269	6536	4	4	0.2163	0.0016
A0A346XM05	TP53 (Fragment)	2	6662	6616	2392	1	0	0.0214	0.0283
Q96FD9	VEGFA protein (Fragment)	25	1232	53955	117495	44	95	0.0008	0.0017

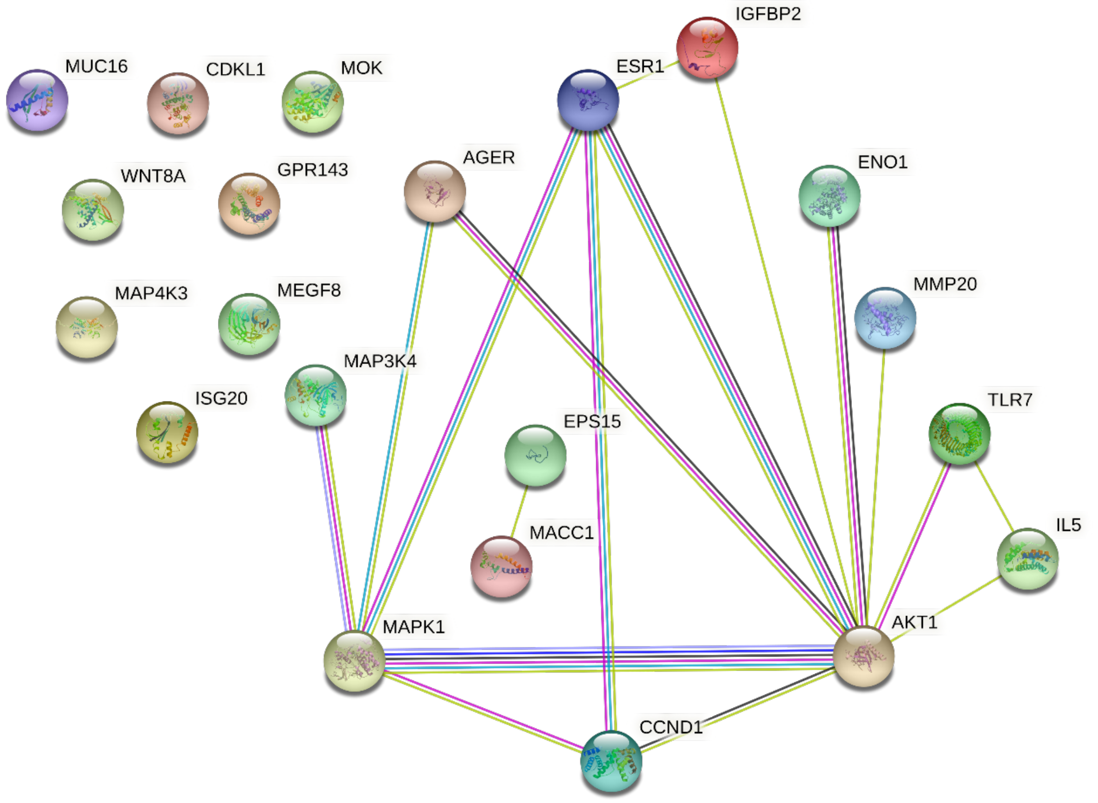

Figure S1 Protein-protein interaction analysis of additional cisplatin/oxaliplatin induced proteins and pro-survival proteins.
